# Exploring Caregiver Challenges And Coping Mechanisms In The Midst Of Covid 19

**DOI:** 10.1093/geroni/igab046.2721

**Published:** 2021-12-17

**Authors:** Devyani Chandran, Marie Eaton

**Affiliations:** 1 Western Washington University, Redmond, Washington, United States; 2 Western Washington University, Western Washington University, Washington, United States

## Abstract

This paper reports on the the challenges faced and coping strategies employed community-based care providers in a mid sized city in the Pacific Northwest in the midst of the Covid 19 pandemic. Researchers from a Palliative Care Institute conducted six online focus groups using the zoom platform. The project aimed at gathering important information on the experiences of caregivers as well as providing a virtual space where caregivers could support each other. A purposive sampling technique was used to gather data where participants were not chosen randomly but because they could best answer the research question. Membership of the focus groups included representatives from skilled nursing facilities, home-care agencies, elder law services, memory-care facilities, adult family homes, medical supply facilities, chaplains and nutritionists. Data gathered from the focus group were transcribed. A constant comparison method of analysis were employed and categories and themes were created from open coded data. Six key themes were identified which included: dealing with the impact of social isolation on caregiver-client relationships, assessing personal risk when dealing with clients living with dementia, facing challenges seeking continuing employment, struggling with social support and self care, using technology for professional and personal support and grappling with the challenges reentering a face to face environment once the pandemic is controlled.. Findings point to the importance developing and sustaining technological innovations that support workforce retention, fostering communication between the larger community, care providers and clients in various care settings and planning for safe reentry into a post Covid world.

